# Can behavioural science be used to understand factors that influence the prescription choice for Parkinson’s disease? A pan-European focus group study of clinicians’ prescribing practice

**DOI:** 10.1136/bmjopen-2024-090018

**Published:** 2025-02-19

**Authors:** Emma Begley, Jason Michael Thomas, William Hind, Carl Senior

**Affiliations:** 1 School of Psychology, College of Health and Life Sciences, Aston University, Birmingham, UK; 2 Alpharmaxim, Altrincham, UK

**Keywords:** Parkinson-s disease, QUALITATIVE RESEARCH, Prescriptions

## Abstract

**Abstract:**

**Objectives:**

This study aimed to establish a consensus on key factors that influence medication choices for Parkinson’s disease and to identify the behavioural determinants of these factors using behavioural change theory as a theoretical lens.

**Design:**

This qualitative study used the nominal group technique to conduct structured online focus group meetings. A facilitator guided participants to (1) individually generate a list of factors that influence their decision to prescribe, (2) collectively share these factors, (3) refine and clarify factors and (4) rank the most important factors. Subsequently, the most important factors identified were mapped to the Theoretical Domains Framework (TDF) and the Capability, Opportunity, Motivation–Behaviour (COM-B) model to identify the behavioural determinants that influence medication choice.

**Participants:**

Eighteen healthcare professionals, including neurologists, consultants and specialist nurses/practitioners who prescribe medication, were recruited across Europe and participated in one of seven focus groups.

**Results:**

There was good consensus among the participants about which factors influence their prescribing decisions. Overall, participants identified 60 unique factors that were broadly categorised into the following themes: medical or symptom concern, patient characteristics, side effects, access to treatment, clinical guidelines, social support and patient preference. Factors discussed and prioritised by the participants aligned with seven of the 14 TDF domains: knowledge; memory, attention and decision processes; beliefs about consequences; goals; social/professional role and identity; environment context and resources; and social influences. Together, these were subsequently mapped onto four of the six subdomains of the COM-B model: psychological capability, reflective motivation, physical opportunity and social opportunity.

**Conclusions:**

These findings suggest that prescribing decisions for Parkinson’s disease are determined by a complex range of factors linked to the COM-B components capability, motivation and opportunity. These can be further understood by specific behavioural domains, as identified by the TDF, which should be targeted to help optimise subsequent prescribing decisions.

**Strengths and limitations of this study**

- Participants were recruited from across Europe, thereby gathering insights from a range of different healthcare environments.
- The structured approach of the nominal group technique was an efficient and effective method that was well received by participants and enabled all participant opinions to be heard.
- The study applied well-used and validated psychological theory to interpret the results.
- Difficulty coordinating participant availability led to a small number of participants per focus group (two or three in each group), which may have reduced the breadth of factors generated.
- The geographic distribution of participants was skewed towards the UK, which may have caused participants from other countries to feel an imbalance when voicing their opinions.

## Introduction

 Parkinson’s disease (PD) is the fastest-growing neurological condition worldwide, which, in part, can be explained by an ageing population who are developing PD and living longer with it.^1^ In 2016, 6.1 million individuals were estimated to have PD, rising to 8.5 million in 2017, and with prevalence rates projected to rise further still, to almost 14.2 million by 2040.^1^,^3^ Consequently, the burden placed on healthcare systems due to PD (eg, cost and medical needs) is also likely to increase. Currently, there is no therapy to cure or slow down the progression of PD, and due to the variability of symptoms, patients often require a personalised management approach that uses the growing repertoire of therapeutic options available.^4^ Levodopa has been used for over 50 years, and it remains the gold-standard treatment for symptoms of PD despite the potential unresolved side effects that can trigger dyskinesia and OFF symptoms in patients.^5^ Medical experts continue to call for new, more effective therapies,^6^ and although some progress has been made (eg, 47 clinical trials exploring PD therapies were registered between 2008 and June 2021), more than 96% of drugs fail during development.^5^ Hence, it is critical that, with the medication available, the best options are prescribed for each patient and, as new more effective drugs arrive, that healthcare professionals (HCPs) are informed in their choices.

It is noteworthy that although clinical reasoning is the foundation of medical practice, there is only limited evidence regarding how these processes are formulated in chronic care^7 8^ (eg, for neurodegenerative diseases, such as PD). It is clear that evidence-based medicine and shared decision-making synergise to help HCPs make decisions; however, balancing patients’ health priorities and their autonomy of treatment preference is still a continuous challenge.^8^ Further, several psychological influences are likely to contribute to clinical decisions. For example, the level of pressure general practitioners (GPs) are working under has been shown to influence their choice of antibiotics; GPs working under increased pressure (eg, high demand from patients, insufficient time and resources, long working hours) prescribed 6.4% more broad-spectrum antibiotics than GPs working under less pressure.^9^ Where there is considerable uncertainty (eg, in the context of multimorbidity management), clinical reasoning may also be influenced by emotions such as fear, anxiety and frustration, leading to hesitancy and clinical inertia.^7^ Clinician bias is another factor that has been reported as influencing which treatments clinicians recommend to patients, further highlighting the importance of understanding the processes behind these clinical decisions.^8^ There is clearly a wide variety of factors that can influence HCP decision-making, and there is a risk of negatively impacting healthcare practice by underestimating the role of these influences.^7^ This is particularly relevant to PD, where there is limited information regarding the factors that might influence prescribing decisions.^10^

Another key consideration is how evidence regarding such factors, if it is available, can be used to impact practice. Theories of behavioural science, such as the Behavioural Change Wheel (BCW) and Theoretical Domains Framework (TDF), offer a robust and transparent way of understanding the determinants of behaviours (eg, prescribing medication for patients with PD) and help identify an effective way to target change.^11 12^ Indeed, the application of behavioural theory has been used previously to explore prescription medicine decisions^13^ and investigate behavioural change interventions to optimise prescribing^14^; however, little is understood about the theoretical determinants that influence medication choices for PD. Changing prescribing behaviours to optimise existing and new drug therapies for PD requires an in-depth understanding of the wide variety of factors that influence HCP decision-making. Moreover, isolating the key factors (ie, those which have the greatest influence on decision-making) is equally important if appropriate targets for behavioural changes are to be identified.

Exploring the lived experiences of HCPs who prescribe medication offers an opportunity to gather valuable data to identify these factors. Hence, this study aims to establish a consensus on the key factors that influence prescription medication choices among HCPs who prescribe medication for PD. A secondary aim is to understand and therefore be able to target the behavioural determinants from a behavioural change perspective to support the optimisation of medication for PD.

## Methodology

### Study design

This study is qualitative and draws on deductive and inductive analysis. Cross-sectional online focus groups were designed and delivered using the Nominal Group Technique (NGT) method to identify and establish a consensus on the factors that influence HCPs’ choice of medication for patients with PD. The NGT method was chosen because it is a highly structured approach that offers a discursive and democratic method of collecting data and creates a collaborative balance among participants.^15^ In comparison to other qualitative research techniques (eg, in-depth interviews), NGT diminishes the potential for facilitator bias and encourages participants to occupy an active, democratically led role. Originally developed to assist in healthcare planning,^16^ the method lends itself particularly well to healthcare research and has been used to develop a framework for care coordination,^17^ explore stakeholder views on hypertension medication adherence,^18^ manage Alzheimer’s disease (using a modified version of the NGT)^19^ and identify research priorities for PD management.^20^ The benefit of the NGT approach is that it is possible to generate ideas, problem solve and establish a consensus that will identify key priorities for a given topic in a timely manner.^20^

Notably, the NGT allows a thematic structure to emerge organically within each group, without imposing any preconceived analytic framework by the facilitator.^21^ The approach taken aligns closely with a critical realist perspective.^22^ Central to critical realism are principles asserting the existence of a multilayered real world shaped by underlying causal mechanisms. These mechanisms generate phenomena, which are then experienced by individuals (eg, how do individual clinicians *interpret* guidelines to make prescription decisions?). However, as these mechanisms are not directly observable due to the complex nature of reality, they are inferred through exploring how people construct and attribute meaning to their experiences of the phenomena (eg, how clinicians *use* guidelines to make prescription decisions).^23^

In the context of prescribing medication for PD, understanding the underlying mechanisms that drive prescribing behaviours becomes crucial. While mechanisms possess the potential to produce phenomena, their causal efficiency is contingent on the contextual conditions within which they operate. This notion of an ‘open system’ acknowledges that the effectiveness of a mechanism can be influenced by other coexisting mechanisms within a given context.^21^

Employing a ‘group-by-group’ critical realist analytical approach allows for the exploration of variation within and between responses, ultimately leading to the identification of themes across the focus groups. The unique strength of this approach is that it can facilitate the between-group comparison of key responses. These themes can serve as valuable indicators of the similarities and differences in how HCPs construct their experiences of prescribing, shedding light on the nuanced factors shaping prescribing behaviours within the complex landscape of PD management.

### Participants and recruitment

Participants were opportunistically recruited and initiated using an online poster disseminated via neurodegenerative disease societies and social network platforms. Experts in the PD field were individually identified using relevant conference/society programmes and websites and, where possible, contacted via email and invited to take part. Snowball sampling was also employed to support further participant recruitment. Participation in the study was open to HCPs from across Europe who spoke English, were currently employed as medical professionals (eg, consultant, specialist nurse, neurologist) and had prescribed medication for PD in the past 2 years (2021–2023). Prospective participants were excluded if they were retired or did not hold a licence to prescribe medication. The recruitment process ran from March 2023 and ended once 18 HCPs had enrolled (and seven groups had been recruited), which is consistent with the critical realist assumptions underpinning the study^22^ and also supported by previous research, which indicates that 80% of data saturation can be achieved with two to three groups.^23^ One participant who agreed to take part left the study before the discussion began, as they were no longer involved in prescribing medication. All participants provided digitally informed consent, thereby indicating that they understood their participation was voluntary and confidential and that they were free to withdraw at any point without having to provide a reason. To thank participants for their time, they were remunerated with a £/€20 online shopping voucher once the focus group meetings were complete. This study received full ethical approval from the Aston University College of Health and Life Sciences Research Ethics Committee, Birmingham, UK, in addition to the NHS Health Research Authority and Care Research Wales Research Ethics Committee (24/HRA/0792).

### Data collection

To ensure the authenticity and depth of insights gathered from the participants, each focus group was facilitated by the lead author (EB), who had no prior contact with the participants, thus minimising the potential for social desirability artefacts to influence the data collection process. Participants had no prior knowledge of the research or of the researcher and only understood the aims of doing the research by reading the participant information sheet. Each of the seven focus groups lasted 1 hour and took place online using Microsoft Teams, with each participant attending only one meeting. Hosting the meetings virtually enabled wider data collection from participants taking part across Europe.^24^ The video of each meeting was digitally recorded for sense-checking purposes but was not transcribed because the focus group consensus of priority factors was the only relevant output data; no participant quotations were used. As well as EB, an assistant was present at the meetings to create field notes that would support later interpretation of the context of the factors discussed.

### Nominal group technique (NGT)

Each focus group commenced with a scene setting (10 min—welcome, aims, purpose, research interest and procedural details) followed by the presentation of the nominal prompt, ‘What factors influence your choice of prescription treatment for Parkinson’s disease?’ to guide subsequent discussion (the prompt was not piloted, nor were participants shown it before taking part). A silent generation phase (5 min) allowed participants to independently scribe responses to the prompt. Each participant then shared their ideas during a round-robin discussion (10 min) facilitated by EB, who compiled a comprehensive list and shared it on screen for participants. During the clarification phase (10 min), participants were encouraged to refine, merge or eliminate duplicate ideas to streamline the list for further assessment. In the subsequent ranking stage (15 min), participants individually ranked their top five factors in order of importance (1=most important; 5=least important) before they were collectively discussed and grouped by the facilitator. Finally, the ranked factors were revealed, allowing participants to collectively agree on the group ranking and address any disparities through discussion or voting for amendments (10 min).

### Data analysis

The NGT approach enables data analysis to take place during the focus group. Data were interpreted qualitatively by allowing a subordinate list of themes to emerge at the round-robin stage of each group and later refined and merged where possible in the clarification stage.^25^ Using these lists, each group agreed on the superordinate ranked factors by voting on their importance. The superordinate ranked factors that emerged from group consensus were then deductively coded to the behavioural domains of the TDF and Capability, Opportunity, Motivation–Behaviour (COM-B) model to identify the behavioural determinants underpinning these factors. This deductive stage involved an iterative process of agreement between authors EB, JT and CS, who coded a sample of the NGT output until the superordinate themes could not be reduced further on to the behavioural domains of the TDF and COM-B models. After meeting regularly to discuss mapping priorities, EB mapped the remaining output into the TDF domains, a process that was iteratively revised with regular discussions with CS. This multistage deductive approach to mapping the behaviours from the focus groups to the specific TDF domains followed the existing work in the field.^26^ A codebook defining each of the TDF domains and how each factor is mapped onto them is provided in online supplemental file 1.

### Research team and reflexivity

Dr Emma Begley PhD is a qualified female researcher with 11 years of experience in public health and behavioural change. At the time of the study, EB was employed as a Knowledge Transfer Partnership Research Associate at Aston University, Birmingham, UK. Dr Jason Thomas and Dr Carl Senior are behavioural scientists with 30 years of experience between them in applying behavioural change techniques in areas such as healthy eating. Any prior knowledge about PD was established during a literature review for EB, JT and CS. William Hind is the Founder and CEO of Alpharmaxim.

### Patient and public involvement

No patients or members of the public were involved in designing or carrying out this research.

## Findings

### Participant characteristics

Across the seven focus groups, a total of 18 participants between 25 and ≥65 years of age took part (female n=10; male n=8). Each focus group comprised two or three participants. Participants’ experience of working in PD ranged from two to ≥20 years as neurologists, consultants, specialist nurses or specialist practitioners (n=5, n=3, n=8 and n=1, respectively; data unavailable for one participant). Most participants were recruited from the UK (n=11); other participants were recruited from Austria, France, Ireland, Italy, Netherlands, Sweden and Switzerland (n=1, from each county).

### Silent generation of factors and round robin (NGT stages 2 and 3)

The raw data that emerged from the silent generation and round-robin stage yielded 60 unique factors that participants thought influenced their prescribing decisions; individual focus groups reported a range of 22–41 factors. There was a good consensus of factors that emerged across the focus groups, and data saturation was reached by the seventh focus group (FG7).

All seven focus groups consistently discussed patient age, HCP and/or patient experience of using a medication, symptoms (including severity and burden) and suitability of a medication (eg, ease of use of medication, route of administration) as important factors. When making prescribing decisions, participants also mentioned how they considered reducing pill burden and simplifying a drug regimen for patients, thereby making it more suitable and acceptable. Other factors commonly reported included consideration of patient cognition (eg, level of education and literacy), complications of comorbidities (eg, hypotension or high blood pressure), side effect profiles, availability of drugs on practice formularies (although participants explained this was not an issue across Austria, Germany or Switzerland), patient quality of life (QoL; eg, daily activity level and ability to still work) and support at home to administer and manage medication.

Drug cost was discussed by all groups, but only five included it in their ranked priorities. There was, however, some discrepancy between participants about whether they considered the cost to be a swaying factor; one participant each from the UK (FG3), France (FG4) and Italy (FG7) expressed that cost was not an influencing factor as efficacy was valued more. There were also inconsistencies regarding the perceived importance of guidelines. Although five focus groups ranked guidelines as being important, either as a factor on its own or as part of a broader theme (eg, external factors, practicalities or drug factors), a few participants (one from FG3 and two from FG5) recalled that they only use them to some extent and preferred to refer to published evidence. Other factors occasionally reported related to polypharmacy, patient and HCP treatment expectations and desired outcomes, patient preference, HCP authority to prescribe, drug efficacy, multidisciplinary team support, psychological health, trials and evidence, capacity to monitor treatment, patient frailty, risk of impulse control disorders and type of PD.

### Clarification and ranking (NGT stages 4 and 5)

Participants ranked between 3 and 8 priority factors; differences in the number of priorities between focus groups occurred due to participant preferences to specify individual factors rather than collapse them into broader themes. As such, the order of rankings is broadly covered (see table 1 for a full overview):

- **Medical or symptom concern** included factors such as the stage of disease, symptom concern or severity and was discussed by six groups, and although four of these groups categorised this factor into a broader theme (disease prognosis, patient characteristics or clinical factors), it was generally ranked a first priority.
- **Patient characteristics** included factors such as patient age, QoL or frailty and was a preferred theme across the groups (five out of seven groups) and was often ranked as a first or second priority, although FG5 ranked it fourth. The two groups that did not include patient characteristics as a superordinate theme decided either to prioritise and individually rank patient age, lifestyle and comorbidity (FG4) or included patient characteristics within a highly ranked ‘clinical factors’ theme (FG7). Despite this variance, the majority of focus groups agreed that factors related to patient characteristics are of high importance when making prescribing decisions.
- **Side effect profile** was discussed by six groups and ranked either as a category by itself or within a broader theme such as response to treatment, practicalities or clinical factors. Three groups ranked this theme as being the third most important. In FG6, it was mentioned that certain medications may be prescribed to manage side effects, but they did not include a side effect profile as one of their ranked themes.
- **Access to treatment**, including availability of treatment, was selected as a priority by four groups, but there was no consistency in its ranking. For example, FG1 ranked this least important (sixth), while FG2, 3 and 6 categorised it into broader themes that were ranked at first, third and fourth priority, respectively. Although the positioning of importance is indifferent between groups, access to treatment was still an important consideration when making medication choices.
- **Clinical guidelines**, including knowledge and practice of, was discussed by six groups, but there was no consensus on ranking between groups. The groups that prioritised guidelines (or included it in broader themes) ranked them first (FG1 and FG2), third (FG3 and FG7) or fourth (FG6). FG5 discussed guidelines but did not prioritise them (see online supplemental file 2), while FG4 did not discuss guidelines at all.
- **Social support**, including carer support at home to manage treatment, was a priority for five groups; three of these groups categorised it into broader themes (eg, patient characteristics [FG1 and FG2], external factors [FG2]). There was no consensus on its ranking, but participants did discuss the importance of having support at home to manage certain treatment.
- **Patient preference** was a priority for five groups: two groups ranked it as a fifth priority, while the other three groups included it within broader themes that were ranked first (FG3) or second (FG1 and 7). Generally, participants discussed the relevance of understanding what patients want and listening to their requests when they have done their own research about the available treatment.

**Table 1:** Factors/categories and ranked priorities

Factors/categories (n, country)	Participant (P) ranking			Final group consensus
**FG1(n=3,UK)**	**P1**	**P2**	**P3**	
Local/national guidelines	6	1	1	1
Patient characteristics	1	2	2	2
Disease prognosis/stage of disease	3	4	3	3
Response to treatment	2	3	4	4
Interpersonal relationships	4	5	5	5
Access to treatment	5	6	6	6
**FG2 (n=3, UK)**	**P1**	**P2**	**P3**	
External factors	1	1	3	1
Patient characteristics	2	2	1	2
HCP experience	3	4	2	3
Side effects	4	3	4	4
**FG3 (n=2, UK)**	**P1**	**P2**		
Patient characteristics	1	1		1
HCP experience	2	2		2
Practicalities	3	3		3
**FG4 (n=3, UK, IRE, FR)**	**P1**	**P2**	**P3**	
Medical concern*	1	1	†	1
Age	3	5	1	2
Side effects	4	2	†	3
Lifestyle	2	†	5	4
Preference	5	3	†	5
Comorbidity	†	†	4	6
Motor ability	†	†	2‡	Not prioritised
Cognition	†	4§	3‡	Not prioritised
**FG5 (n=2, NL,SE)**	**P1**	**P2**		
Symptoms	1	1		1
Efficacy and safety	3	2		2
Side effects	4	3		3
Patient characteristics	2	4		4
Preference	5	5		5
**FG6 (n=3, UK, IT)**	**P1**	**P2**	**P3**	
Patient characteristics	1	1	1	1
Quality of life	2	2	2	2
Social setting	3	3	3	3
Drug factors	5	4	4	4
Prescriber experience/culture	4	5	5	5
**FG7 (n=2, IT, CH)**	**P1**	**P2**		
Clinical factors	1	1		1
Shared decision-making	3	3		2
Guidelines	2	4		3
Treatment needs	4	2		4
Cost	5	†		5¶
Social support	†	5		5¶

* Participants in this group had differing opinions about whether patients’ cognitive ability and degree of motor symptoms should be included within this category or ranked separately (see footnotes c‡ and d§).

† pParticipant did not rank this factor.

‡ pParticipant ranked both ‘Motor’ and ‘Cognition’ separately in positions 2 and 3, respectively.

§ pParticipant ranked ‘Cognition’ separately in position 4.

¶ pParticipants could not reach a consensus on which factor should be ranked in position 5. They decided that it was country-dependent so recommended different factors for CH and for ITLY.

AT, Austria; CH, Switzerland; FG, focus group; FR, France; HCP, healthcare professional; IRE, Ireland; NL, Netherlands; P, participant; SE, Sweden; IT, Italy; UK, United Kingdom

Other factors that were prioritised and ranked but could not be easily grouped into the above themes included interpersonal relationships, such as multidisciplinary support (ranked fifth by FG1); efficacy and safety of a drug (ranked second by FG5, while three other focus groups included it within broader themes); treatment needs, such as patient expectation and symptom treatment (ranked fourth by FG7); and cost (ranked fifth by FG7, and included within broader themes for four other groups; however, some participants did not perceive cost as a swaying factor).

### Theoretical mapping of priorities

**Figure 1:**
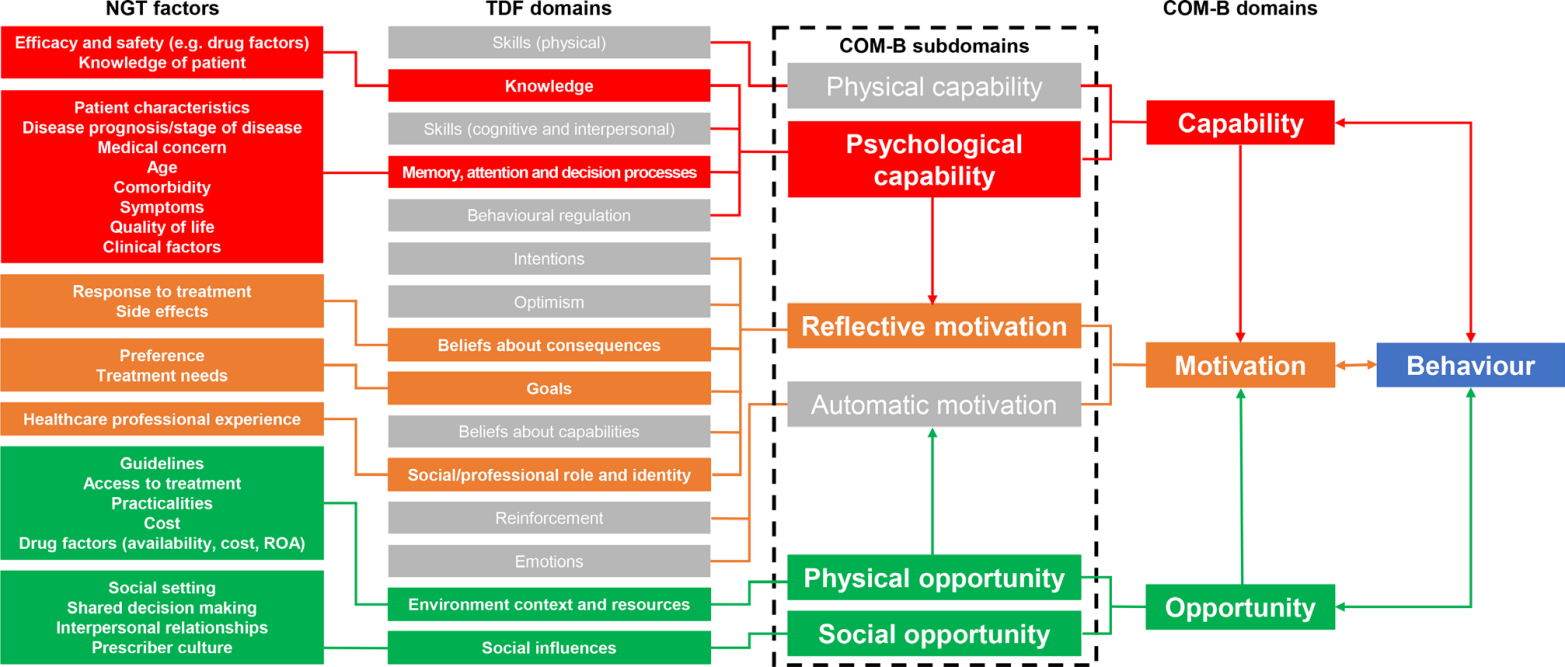
Mapping of priority factors onto TDF and COM-B domains. Red blocks: factors mapped to the capability COM-B domain; orange blocks: factors mapped to the motivation COM-B domain; green blocks: factors mapped to the opportunity COM-B domain. The grey boxes are not applicable to the current data set and are included here for reference only. COM-B, Capability, Opportunity, Motivation—Behaviour; DDI, drug–drug interaction; NGT, nominal group technique; ROA, route of administration; TDF, Theoretical Domains Framework.

Mapping priorities to the TDF provides a structured method to uncover the various behavioural determinants that may influence prescribing outcomes. This approach can help tailor evidence-based strategies to the unique context of that behaviour, increasing the likelihood of successful and sustainable change. To achieve this, the factors discussed and prioritised by participants aligned with seven of the 14 TDF domains, which subsequently mapped onto four of the COM-B subdomains (see figure 1 below for a mapping overview). Prioritised factors from the focus groups aligned with the following two TDF domains: knowledge and memory, attention and decision processes, which themselves map onto the COM-B capability domain (specifically, the psychological subdomain). Most of the factors mapped onto these domains were linked to HCP decision-making processes and were informed by patient and clinical characteristics: factors that participants ranked as being the most important. In terms of knowledge, participants discussed that their understanding of the efficacy and safety of a drug, including the interaction of other medication, influenced their prescribing (see description of factors in online supplemental file 2).

Another suite of prioritised factors aligned with the following three TDF domains: belief about consequences, goals and social/professional role and identity, which map onto the COM-B motivation domain (specifically, the reflective subdomain). Participants often shared concerns about the consequences of a medication causing side effects, such as reduced impulsive control and the potential of medication to negatively impact patient QoL, or reduce or impair treatment outcomes. As such, participants discussed how it was important for them to consider patient preferences and identify treatment needs, subsequently informing treatment goals. Participants also felt that HCP experience and familiarity with prescribing a medication would influence future prescribing decisions (see online supplemental file 2); this was particularly evident among specialist nurses who explained how they often discuss prescribing options with senior consultants or neurologists.

Finally, the remaining prioritised factors from the focus groups aligned with the following two TDF domains: environment context and resources, and social influences, which map onto the COM-B opportunity domain (the physical and social subdomains, respectively). Factors within these domains reflected influences arising from the participants’ physical environment (eg, medication cost or availability) and resources (eg, guidelines or access to medication) that somewhat determined prescribing decisions. Additionally, a range of social influences, such as the interpersonal relationships that arise from shared decision-making with patients or HCP peers and support at home to help patients manage medication and a general prescribing culture, was reported by participants.

## Discussion

This study provides a coherent consensus of a variety of key factors that influence prescription medication choice for PD (first aim) and a theoretically informed understanding of the behavioural determinants that underpin HCP decisions (second aim). First, the NGT focus groups identified 60 unique factors that influence HCP prescribing medication choices for PD. Eighteen participants highlighted that medical or symptom concerns (often grouped into patient characteristics), followed by medication side effects, were consistently considered as important factors that influenced medication choices. Several additional factors (access to treatment, clinical guidelines, social support, patient preference, interpersonal relationships, efficacy and safety, treatment needs and cost) were also important; however, there was no consistency in their ranking. Second, seven TDF determinants were mapped to these factors and assessed using the COM-B model, which indicated that an HCP’s psychological capability, physical and social opportunity and reflective motivation are important determinants when making medication choices.

The factors presented in this study are consistent with those reported by existing research. For example, a systematic review of 44 studies found that patient age was the most common factor that influenced prescription medication for PD.^10^ Reasons for this included concerns regarding side effects, drug interactions or increased morbidity in older adults,^10^ factors that were also ranked as important in this study. Previous research also indicated that choosing a medication that facilitates an improved QoL for patients is often a priority,^27 28^ which may explain why participants often discussed patient-directed goals (eg, desire to still work) and treatment preferences in their ranked priorities. Issuing patients with their preferred treatment may, however, be hindered by time pressure, a barrier that has notably affected the provision of preventive health services in other therapeutic areas.^29 30^ The dichotomy of HCP opinion on the influence of guidelines is also worthy of discussion; this occurred mostly between specialist HCPs (eg, consultants or neurologists) who referred less to them and other medical prescribers (eg, specialist nurses or GPs) who ranked them as important. This inconsistency of HCP adherence to guidelines is commonly reported,^10^ and the available evidence suggests that barriers to guideline adherence or evidence-based medicine may be due to HCPs' lack of time,^30^ increased pressure^9^ or previous experiences and patient preference for certain medication.^31^

By identifying these factors and understanding them in behavioural terms (ie, what theoretically facilitates or is a barrier to performing a behaviour), it enables intervention designers to develop a robust strategy that more effectively brings about a desired change in behaviours.^11 32^ The effectiveness of doing so is reflected in a review that also used the TDF to identify behavioural determinants from interventions addressing medication optimisation more broadly.^14^ The review identified 16 effective interventions that used a variation of nine TDF determinants to optimise medication prescribing; however, the authors noted that not all interventions used the array of behavioural change techniques needed to target the determinants identified.^14^ Still, it is encouraging that the nine determinants reported in the review encapsulate all of the seven determinants reported in this current study, and the review provided some indication of which behavioural change techniques (ie, prompts and default options) could be used to optimise prescribing.^14^ Furthermore, knowledge of the COM-B domains identified in this study means that the latter steps of the BCW (eg, identifying intervention functions and behavioural change techniques) can be conducted to identify other potential behavioural change techniques to optimise the prescribing choice.

Reflecting on the present study, a notable strength is that participants were opportunistically recruited from across Europe, thereby gathering insights from a range of different healthcare environments. A further strength in the study design was that a comparison was possible across several focus groups, thereby contributing to the reliability of the data. The structured approach of the NGT was also well received by participants; it was viewed as an efficient and effective method that heard all participants’ opinions. It is also a strength that the study applied well-used and validated psychological theory to interpret the results. The ease in which it was possible to segment the behavioural themes across a European sample was also a clear strength of this study.

However, this study is not without limitations. The number of participants per focus group ranged between two and three; this was largely due to the difficulty in coordinating participant availability. As a result, lower focus group numbers may have reduced the breadth of factors generated. The geographic distribution of participants was also skewed towards the UK; as such, participants from other European countries with different cultural views may have felt an imbalance when voicing their opinion. Hence, future work in this field might look to use different methodologies to test a wider range of individuals, in such a way as to reduce the effect of group dynamics on response (eg, a large, multi-country quantitative survey focused on the factors identified within this study).

In conclusion, the factors that influence HCPs’ choice of medication for PD are clearly multifaceted, and the evidence presented here indicates that prescription decisions are primarily driven by clinical presentation and patient characteristics. To optimise the treatment that patients with PD are prescribed, behavioural interventions should consider approaches that collectively target HCPs’ psychological (eg, knowledge) capability, physical (eg, availability of drugs) and social (eg, peer influence) opportunities and reflective (eg, reduce beliefs of potential consequences) motivation. When developing an intervention to target optimised prescribing for PD, it will be important to create a strategy that will address each of these behavioural determinants. It is also clear that there is utility in this approach for the study of prescription behaviours in other disease states. The study reported in this paper forms part of a broader programme of research currently being undertaken which aims to address key challenges in clinical decision-making. The aim of the current work is to understand consensus on behaviour around prescribing which will serve to allow us to highlight the ways in which it can, ultimately, be improved.

## supplementary material

10.1136/bmjopen-2024-090018 online supplemental file 1

10.1136/bmjopen-2024-090018 online supplemental file 2

## Data Availability

All data relevant to the study are included in the article or uploaded as supplementary information.
