# Supplementary material for: Can behavioural science be used to understand factors that influence the prescription choice for Parkinson’s disease? A pan-European focus group study of clinicians’ prescribing practice
Source: BMJ Open. 2025 Feb 19;15(2):e090018. doi: 10.1136/bmjopen-2024-090018 (PMC11840894; doi:10.1136/bmjopen-2024-090018)
Supplement: online supplemental file 1 [file bmjopen-15-2-s001.docx]

Supplemental material Table 1. TDF coding for NGT.

| **TDF domains (Cane, et al. 2012)** | **Original definition** | **Coding rules** | **COM-B (Michie, et al. 2011)** |
| --- | --- | --- | --- |
| Knowledge | An awareness of the existence of something | Reference to already having knowledge, lack of knowledge or resource of knowledge, e.g., guidelines/scientific literature | Capability |
| Skills | An ability or proficiency acquired through practice | Reference to having or attending specific training courses or experience of practice. |  |
| Behavioural Regulation | Anything aimed at managing or changing objectively observed or measured actions | Discussion of checklists or prompts served as a reminder to change prescribing |  |
| Memory, attention and decision making | The ability to retain information, focus selectively on aspects of the environment and choose between two or more alternatives | Biopsychosocial patient factors that inform decision making/action, recollection of physician knowledge, concentration/attention to tasks |  |
| Social/professional role and identity | A coherent set of behaviours and displayed personal qualities of an individual in a social or work setting | Self-perceived level of specialism or defined professional role | Motivation |
| Beliefs about capabilities | Acceptance of the truth, or validity about an ability, talent or facility that a person can put to constructive use | Recognised competency or self-efficacy to perform/manage certain tasks. |  |
| Optimism | The confidence that things will happen for the best or that desired goals will be attained | Expectation of positive outcomes |  |
| Beliefs about consequences | Acceptance of the truth, or validity about outcomes of a behaviour in a given situation | Reported beliefs of potential negative outcomes, the likelihood of increased risk or fallacious beliefs |  |
| Reinforcement | Increasing the probability of a response by arranging a dependent relationship or contingency between the response and a given stimulus | Discussion around having a positive impact that encouraged prescribing behaviour |  |
| Intentions | A conscious decision to perform a behaviour or a resolve to act in a certain way | Assertive decision or plan to act |  |
| Emotion | A complex reaction pattern, involving experiential, behavioural and physiological elements, by which the individual attempts to deal with a personally significant matter or event | Reports of an evoked emotional reaction such as anxiety, fear, stress or distress that influences prescribing |  |
| Goals | Mental representations of outcomes or end states that an individual wants to achieve | Discussion of a desirable aim or achievable outcomes |  |
| Environmental context and resources | Any circumstance of a person’s situation or environment that discourages or encourages the development of skills and abilities, independence, social competence and adaptive behaviour | Discussion around convenience of administrating medication, cost, access, environmental setting or geographical residency | Opportunity |
| Social influence | Those interpersonal processes that can cause individuals to change their thoughts, feelings or behaviours | Peer, carer, patient preference or opinion that influences a decision; additionally, changing guidelines, media exposure and discussion around prescribing cultures |  |
