# Supplementary material for: Can behavioural science be used to understand factors that influence the prescription choice for Parkinson’s disease? A pan-European focus group study of clinicians’ prescribing practice
Source: BMJ Open. 2025 Feb 19;15(2):e090018. doi: 10.1136/bmjopen-2024-090018 (PMC11840894; doi:10.1136/bmjopen-2024-090018)
Supplement: online supplemental file 2 [file bmjopen-15-2-s002.docx]

Supplemental Material Table 1. Description of factors and themes as discussed by participants and the corresponding TDF determinants.

| Refined factors/categories | Description of factors | TDF (COM-B) determinates |
| --- | --- | --- |
| **Focus group 1** | | |
| Local/national guidelines | National Institute for Care Excellence (NICE) guidance | Knowledge (psychological capability) |
| Patient characteristics | Age, cognition, comorbidities, mood/mental health, blood pressure, gastric quality, ability to swallow, patient experience of using a drug, patient choice, polypharmacy, physical wellness, route of administration, ability to adhere to medication advice, home support | Memory, attention and decision processes (psychological capability) |
| Disease prognosis | Distinguishing disease factors: stage of disease, duration of condition, diagnosis, shock of diagnosis, symptoms of tremor, severity of symptoms, guidelines | Memory, attention and decision processes (psychological capability) |
| Response to treatment | QoL, aligns with patient and HCP outcome expectation, ability/ease to objectively measure positive response to treatment/desired outcome | Belief about consequences (reflective motivation) |
| Interpersonal relationships | HCP experience of prescribing a treatment, advice from consultant, responsibility of the HCP to prescribe, specialist approval, access to MDTs or peer advice | Social influences (social opportunity) |
| Access to treatment | Cost, availability of medication in blister packs, ease of use, availability of medication in practice formulary | Environment context and resources (physical opportunity) |
| **Focus group 2** | | |
| External factors | NICE guidelines, new research, the media, treatment availability, waiting times for treatment, positive or negative HCP or peer experience of prescribing medication, HCP and patient support using the medication, cost and availability | Environment context and resources (physical opportunity)  Social/professional role and identify (reflective motivation)  Social influences (social opportunity) |
| Patient characteristics | Age, biological age, patient condition (e.g., their cognitive state and suitability to receive a medication), comorbidity, psychiatric history, suitability of route of administration of medication, polypharmacy | Memory, attention and decision processes (psychological capability) |
| HCP experience | HCPs personal experience of prescribing a medication | Social/professional role and identify (reflective motivation) |
| Side effects | Medication side effects | Belief about consequences (reflective motivation) |
| **Focus group 3** | | |
| Patient characteristics | Age, comorbidities, stage of disease, sub-type of PD, symptoms and symptom severity, impact on QoL, social support, side effect profile, patient choice, patient expectation and desired outcome of treatment | Memory, attention and decision processes (psychological capability) |
| HCP experience | Personal clinical experience, clinical judgement, familiarity with medication, knowledge of efficacy, opportunity and knowledge of clinical trials | Social/professional role and identify (reflective motivation) |
| Practicalities | Ease of medication administration, efficacy of treatment for symptoms, side effect profile, capacity to monitor patients, availability of medication in local formulary, cost, guidelines | Environment context and resources (physical opportunity)  Belief about consequences (reflective motivation) |
| **Focus group 4** | | |
| Medical concern | Patient and HCP treatment concerns, consider patients cognitive ability and motor *versus* non-motor symptoms | Memory, attention and decision processes (psychological capability) |
| Age | – | Memory, attention and decision processes (psychological capability) |
| Side effects | Side effects from previous medication, history of impulse control, consideration that medication does not affect current lifestyle | Belief about consequences (reflective motivation) |
| Lifestyle | Employment status, daily activity, PKG report, social support and family | Memory, attention and decision processes (psychological capability) |
| Preference | Patient expectation and desired treatment outcome, adherence, route of administration, patient requests | Goals (reflective motivation) |
| Comorbidity | – | Memory, attention and decision processes (psychological capability) |
| Factors discussed but not prioritised: access to multidisciplinary team, cost, drug availability | | |
| **Focus group 5** | | |
| Symptoms | Severity of symptoms, impact on daily life | Memory, attention and decision processes (psychological capability) |
| Efficacy and safety | Efficacy of medication, safety of use | Knowledge (psychological capability) |
| Side effects | Risk of side effects, side effect profile, side effects experienced from previous medication | Belief about consequences (reflective motivation) |
| Patient characteristics | Age, gender, weight, cognition, patient education | Memory, attention and decision processes (psychological capability) |
| Preference | Patient choice, request | Goals (reflective motivation) |
| Factors discussed but not prioritised: HCP personal experience of prescribing a medication, guidelines to an extent, local tradition, cost, cost reimbursement, availability of medication, adherence | | |
| **Focus group 6** | | |
| Patient characteristics | Age, frailty, comorbidity, symptom burden, symptoms, cognition, education and literacy, past medical history, experience with previous treatment, family member experience, risk of impulsive behaviour, formulation preference, allergies, intolerances, religious beliefs, vegetarian, concordance | Memory, attention and decision processes (psychological capability) |
| Quality of life | Employment status, health beliefs, goals and expectations | Memory, attention and decision processes (psychological capability) |
| Social setting | Home support, current lifestyle, ability and access to follow up with patients | Social influences (social opportunity) |
| Drug factors | Availability of medication, cost, efficacy, pill burden, mode of action, mode of administration, drug-drug interaction, NICE guidelines, using off licence or for side effect/similar | Knowledge (psychological capability) |
| Prescriber experience/culture | Prescriber experience and confidence prescribing a medication, cultural beliefs about prescribing, off-licence use, organisational constraints on prescribing, HCP knowledge of patient | Social influences (social opportunity)  Belief about capabilities (reflective motivation)  Knowledge (psychological capability) |
| **Focus group 7** | | |
| Clinical factors | Type of PD, age, polypharmacy, years since onset, severity of symptoms, side effects, use of previous treatment, comorbidities (e.g., cognitive disorders, hypotension, depression) | Memory, attention and decision processes (psychological capability) |
| Shared decision making | Consideration of what patient wants | Social influences (social opportunity) |
| Guidelines | Treatment guidelines, drug regulation and published evidence of drug efficacy | Environment context and resources (physical opportunity) |
| Treatment needs | Simplify drug schedule, patient expectations of treatment outcome, patient QoL, symptoms patient wants to target | Goals (reflective motivation) |
| Cost | – | Environment context and resources (physical opportunity) |
| Social support | – | Social influences (social opportunity) |
