# Supplementary material for: Can behavioural science be used to understand factors that influence the prescription choice for Parkinson’s disease? A pan-European focus group study of clinicians’ prescribing practice
Source: BMJ Open. 2025 Feb 19;15(2):e090018. doi: 10.1136/bmjopen-2024-090018 (PMC11840894; doi:10.1136/bmjopen-2024-090018)
Supplement: Supplementary file 3 [file bmjopen-2024-090018.reviewer_comments.pdf]

PEER REVIEW HISTORY

BMJ Open publishes all reviews undertaken for accepted manuscripts. Reviewers are asked to complete a checklist review form and are provided with free text boxes to elaborate on their assessment. These free text comments are reproduced below.

ARTICLE DETAILS

Title (Provisional)

Can behavioural science be used to understand factors that influence prescription choice for Parkinson's disease? A pan-European focus group study of clinicians' prescribing practice.

Authors

Begley, Emma; Thomas, Jason Michael; Hind, William; Senior, Carl

---

VERSION 1 - REVIEW

Reviewer 1

Name Ramaswamy, Bhanu

Affiliation Sheffield Hallam University, College of Health, Wellbeing and Life Sciences

Date 21-Oct-2024

COI None

This article contributes to constructs and concepts justifying further the need for a personalised medicine approach to prescribing. A very interesting point made illustrating some decision-making differences between prescribers from a medical background as opposed to those prescribers (like myself) from an allied background. The recruitment of practitioners from countries other than the UK detract from the study somewhat because of the small numbers, but enable discussion to be commenced regarding the differences in practice in different health systems.

Reviewer 2

Name Long, Janet

Affiliation Australian Institute of Health Innovation, Australian Institute of Health Innovation

Date 25-Oct-2024

COI None

This study used a convenience sample of European health professionals currently involved in care of patients with Parkinson's Disease to determine consensus opinion on factors important in clinical decision-making around prescription of PD medications. Nominal group technique was used to elicit and rank factors. The rationale in the introduction framed this as a study around advancing our understanding of clinical decision making around medication prescription in chronic and complex conditions.

The next step in the study is not clearly described and I found illogical. The factors are applied somehow to the Theoretical Domains Framework to come up with "behavioural determinants" suggesting these can be used to design interventions. It is not clear what behaviours nor why they need an intervention.

There is value in the first part of the study, bearing in mind you are mapping current practice rather than best practice however, how each factor is used / applied was not rigorously collected. Lack of this data means the TDF exercise seems inappropriate.

Major issues:

The nominal group exercise elicited consensus opinion on factors important in decision-making. This was not compared to, nor was any comment made about, the quality of the ranked factors. Could you compare them to the guidelines, literature or material from consumer advocacy groups that talk about the patients' priorities?

There is no case made for PD prescribing being sub-optimal or even that it makes a difference to patient's QOL. Anecdotally I know it does but this aspect was not made really clear.

Likewise, there was no case made for the systematising or identification of key factors that should be considered when prescribing for PD other than it is not well understood. Should be linked back to patient outcomes.

The TDF part of the work was: (a) not well explained, (b) not given a clear rationale for inclusion and (c) had underwhelming results.

In the Methods, different sets of concepts were described which I found confusing at times – critical realism describing "mechanisms and contexts", and theoretical domains describing behaviours and "behavioural determinants". I was very confused from this how the TDF was being used and on what data. How did you get from a list of factors to barriers to behaviour? What behaviour? In the Table you have comments on the factors – are they what the participants said or what you interpreted from their comments or a mix of both? Is this reliable enough data?

The TDF does not map "determinants" as such but is used to categorise barriers to performing the defined target behaviour. You do not describe how you define the target behaviours. Are you saying each priority factor is linked to a different target behaviour? If so, how did you identify those behaviours? And what evidence do you have that these behaviours are not being done by the HCPs (implied by talk of intervention development)?

Results of the TDF portion were underwhelming and contributed little to the field.

Consider rewriting the paper to emphasize the ranked factors part of the work and 2consider submitting as a short report. I appreciate how much work went into the TDF section but it doesn't work here with your current data.

VERSION 1 - AUTHOR RESPONSE

Reviewer: 1

This article contributes to constructs and concepts justifying further the need for a personalised medicine approach to prescribing. A very interesting point made illustrating some decision-making differences between prescribers from a medical background as opposed to those prescribers (like myself) from an allied background. The recruitment of practitioners from countries other than the UK detract from the study somewhat because of the small numbers, 3nablee discussion to be commenced regarding the differences in practice in different health systems.

Thank you for your positive comments on our report. We are delighted that you found our work has raised interesting points regarding decision-making differences when it came to prescribing behaviours. Like you, we feel that our work will be of great interest both to clinicians with a medical background and to colleagues from the allied health professions.

With regards to your point:

'The recruitment of practitioners from countries other than the UK detract from the study somewhat because of the small numbers, but enable discussion to be commenced regarding the differences in practice in different health systems.'

It is important to highlight that comparing the outcomes from different Nominal Group Technique (NGT)-based focus groups is a useful approach to identify trends or patterns across the groups. Intentional group segmentation, as was carried out here, helped us to clarify how different prescribers across Europe prioritise decision-making strategies. Indeed, by carrying out this approach, the focus group data enabled us to see distinct strategies emerge (see also the point that we raise in response to R2s comments below). The unique nature of the NGT approach is such that large groups of participants are not required as would be the case with traditional focus-group formats. That said, we are cognisant of the fact that some may perceive the participant numbers to be low. We have already highlighted this as a potential limitation (see 'Strengths and limitations of this study'). We have also taken the opportunity to further include a statement as to the strength of the group segmentation approach and how this contributes to the reliability of the data in the latter aspects of the discussion section of our report (see para 4, 'Discussion').

Reviewer: 2

Dr. Janet Long, Australian Institute of Health Innovation
Comments to the Author:

Thank you very much for your consideration of our report which we feel has been significantly improved with the constructive comments from both yourself and R1. We are

delighted that you highlighted the key points of our work as reported here and have addressed each of your comments below on a point-by-point basis. As you will see, we have also taken this opportunity to strengthen the link to the TDF model in our report, which will enable the readership to clearly see the value of this theoretical model to understanding the factors that drive prescription behaviours across Europe. We are in no doubt that that this revision has strengthened our report and thank you for highlighting this opportunity for us.

This study used a convenience sample of European health professionals currently involved in care of patients with Parkinson's Disease to determine consensus opinion on factors important in clinical decision-making around prescription of PD medications. Nominal group technique was used to elicit and rank factors.

The primary objective of this study was to achieve a comprehensive understanding of the factors influencing prescription decision-making, and we are pleased to have accomplished this goal. The pan-European nature of the sample was specifically chosen to facilitate the development of a robust framework, paving the way for a larger and more detailed exploration of the variables shaping prescribing decisions across diverse healthcare systems and cultural contexts. By including participants from various regions of Europe, the study ensured a representative and inclusive foundation for these findings.

As is noted above, the NGT approach that we employed here proved instrumental in identifying a cohesive set of themes. This heavily structured approach allowed for the systematic capture and prioritisation of insights directly from the participants, ensuring clarity and depth in the analysis. Once these themes were established, they were contrasted and compared across all focus groups. This comparative analysis was critical for identifying a consistent set of themes relevant to prescribers throughout Europe. The process not only highlighted shared decision-making factors but also illuminated subtle regional variations, ensuring the findings are both universally applicable and contextually nuanced. Our unique analytic approach lays the groundwork for future studies, aiming to deepen understanding and inform policy and practice in European prescription decision-making. We would also like to direct your attention to our response to the comment raised by R1 where we describe the strength of the NGT approach in general and the significant utility realised by cross referencing the outcome of several focus group discussions carried out across Europe.

The rationale in the introduction framed this as a study around advancing our understanding of clinical decision making around medication prescription in chronic and complex conditions.

The study reported in this paper, as well as the broader programme of research currently being undertaken in collaboration with our industry partner and scientific collaborator, Alpharmaxim, aims to address key challenges in clinical decision-making. As is widely acknowledged, clinical prescription behaviours often exhibit a degree of rigidity, a phenomenon cognitive psychologists describe as 'functional fixedness.' While such steadfastness can be beneficial in ensuring consistency and adherence to best practices in many aspects of clinical care, it may also limit opportunities for more adaptable and innovative approaches, particularly in prescribing treatments. This study seeks to explore this dynamic in the context of Parkinson's disease, a condition that offers a compelling case study due to its reliance on a foundational treatment approach centred around the dopamine replacement agent, Levodopa. This has long been considered a cornerstone in the treatment of this condition and occupies a dominant position in the clinical decision-making process. However, the rigid reliance on this treatment approach may inadvertently constrain consideration of alternative or supplementary therapies that could potentially enhance patient outcomes.

By examining the behavioural factors that may reinforce cognitive flexibility in this area, our work can contribute to the reduction of the primacy of a particular treatment modality. The findings presented in the current report aim to encourage clinicians to adopt a more nuanced and flexible approach to prescription behaviours, balancing established practices with the potential of new interventions that should ultimately improve therapeutic outcomes.

The next step in the study is not clearly described and I found illogical.

We sincerely apologise for the somewhat unclear narrative presented in this section and would like to assure you that we have made significant revisions to improve its clarity and coherence. Recognising the importance of providing a transparent and logical progression, we have taken the opportunity to refine this section to ensure that the connection between the collection of the data and its broader implications is unmistakable. These changes are aimed at enhancing the readability and ensuring that the readership can clearly appreciate how these findings align with and contribute to the larger Theoretical Domains Framework. We are now confident that the revised section effectively demonstrates the relevance and value of embedding these findings within this broader theoretical context (see the 'Data analysis' and 'Theoretical mapping of priorities' sections).

The factors are applied somehow to the Theoretical Domains Framework to come up with "behavioural determinants" suggesting these can be used to design interventions. It is not clear what behaviours nor why they need an intervention.

The focus group discussions were conducted using a carefully designed approach aimed at eliciting factors influencing clinical decision-making around prescribing behaviour. Central to the unique NGT approach was the nominal prompt, which guided participants to concentrate on a specific question (see methods). For this study, the prompt (see ' The nominal group technique (NGT)' section) was, "What factors influence your choice of prescription treatment for Parkinson's disease?" As detailed in the 'Findings' section, this method proved highly effective in uncovering themes unique to the cultural and geographical contexts of the participating groups. Notably, as highlighted in the 'Silent generation of factors and round robin (NGT stages 2 and 3)' section, the presence of concordant factors across groups, regardless of their geographical location, underscores the effectiveness of this approach in identifying fundamental determinants of prescribing behaviour in Parkinson's disease.

The mapping of these elicited factors to Theoretical Domains Framework (TDF) domains is detailed in Table 1, explaining how the themes initially identified through the focus groups were aligned with TDF domains. Additionally, the subsequent mapping of TDF domains to the COM-B model, where key behavioural determinants are housed, is described in 'Data analysis'. This section outlines the deductive process used to validate the primary behavioural determinants identified. Further information on the methodological rigor applied in this mapping process can be found in Supplementary File 1 (see also the responses made to R1 above where we further highlight the strengths of the NGT approach as well as the introductory sections of the report which includes a rationale for the study). Finally, figure 1 maps the behaviours that were revealed from the NGT focus groups to the relevant domains of the TDF and COM-B model.

There is value in the first part of the study, bearing in mind you are mapping current practice rather than best practice however, how each factor is used / applied was not rigorously collected. Lack of this data means the TDF exercise seems inappropriate.

Thank you for noting the value that our work has in the field, and we also feel that the readers of the journal will also agree with you and recognise its value. The Nominal Group Technique (NGT) is indeed a more rigorous method of data collection than traditional focus

group formats due to its structured and systematic approach (see also our replies to R1s comments). Unlike traditional focus groups, where discussions can be influenced by group dynamics, power imbalances or dominant personalities, NGT minimises these issues by ensuring that all participants contribute individually and equally at various stages of the process. As is noted in the report, participants first generate ideas independently, reducing the risk of groupthink and bias that can occur when individuals are influenced by the opinions of others. Following this, the ideas are shared, discussed, and then ranked or voted on, providing a clear, quantifiable measure of participants' preferences or priorities. This systematic approach enhances the reliability and validity of the data collected, as it captures a broader range of perspectives and offers a more objective means of synthesising group input, making it a more rigorous and trustworthy method of gathering qualitative data.

The structure of the NGT discussion ensures that all participants' voices are heard and valued. In a traditional focus group, group dynamics can sometimes lead to dominant voices overshadowing quieter participants, which can result in valuable insights being missed. In contrast, NGT provides a structured process where each participant has an equal opportunity to contribute. Participants initially generate ideas individually, then share them with the group in a round-robin format, ensuring a more balanced distribution of input. This method prevents the conversation from being dominated by a few individuals, allowing for a broader range of perspectives to be considered.

By adopting the NGT approach, we have facilitated a more objective and efficient decision-making process. After the ideas were generated and discussed, participants independently ranked or vote on them, this helps to prioritise the most significant ideas based on collective input rather than group consensus alone. This further eliminates the potential for groupthink or peer pressure, where participants might conform to the majority opinion out of social influence. The systematic nature of NGT encourages critical thinking and reduces the influence of dominant personalities, making it particularly valuable in situations where a precise, democratic decision or understanding of diverse opinions is needed. In comparison to traditional focus groups, which may lack this level of structure, NGT is a more effective method to fostering a comprehensive, fair, productive and highly reliable discussion.

The utility of this approach ensues that it is possible to identify a range of ecological valid behaviours that are used by a small cohort of prescribers which can then be used to develop a framework that can be applied for the understanding of behaviours in the larger population – the aim of our report was indeed to capture the 'lived experience of HCPs who prescribe medication' ('Introduction') and <u>not</u> just best practice regarding prescribing behaviour.

Major issues:

The nominal group exercise elicited consensus opinion on factors important in decision-making. This was not compared to, nor was any comment made about, the quality of the ranked factors. Could you compare them to the guidelines, literature or material from consumer advocacy groups that talk about the patients' priorities?

As noted above, the unique approach we adopted here offers several advantages that make it superior to traditional focus group approaches, particularly for the purposes of this study. During the initial design phase, we considered various methodologies but ultimately selected NGT for three fundamental reasons: its efficacy, its independence from preconceived analytical frameworks, and its emphasis on participant expertise.

The efficacy of the approach was instrumental in enabling us to conduct focus groups across multiple countries efficiently. This method allowed us to gather data in a relatively short time frame without sacrificing depth or quality. The unique structure of NGT facilitated this

expedited process, as noted in our report. Second, unlike approaches that rely on predefined analytical frameworks, the NGT method permits the generation of data that is not constrained by existing theoretical constructs. While such frameworks can be beneficial for aligning results with established theories, they were not suitable for our objectives. By avoiding these limitations, we ensured that our findings emerged naturally from the participants' input, free from external bias. Third, at the core of the NGT approach is the recognition that participants are the true experts in their respective fields. This perspective aligns with a critical realist viewpoint, which prioritises capturing authentic, ecologically valid data reflective of everyday behaviours. In our study, this was particularly significant for exploring behaviours related to the generation of prescriptions in neurodegenerative disorders. The quality of the data is evident in the fact that the participants – prescribers themselves – judged the insights to be both relevant and meaningful.

To highlight the critical importance of this methodological choice, we have explicitly included a statement in the 'Methodology' and 'Discussion' sections of our report, ensuring readers understand how the NGT approach was central to achieving the study's goals and capturing reliable, actionable data.

There is no case made for PD prescribing being sub-optimal or even that it makes a difference to patient's QOL. Anecdotally I know it does but this aspect was not made really clear.

Thank you for highlighting this important point, which we believe connects to a much broader discussion concerning the nature of prescribing practices in and around Parkinson's disease. Although we did not make any explicit initial claims regarding the efficacy of prescribing for this patient population, your observation raises an interesting concern. Anecdotally, there could indeed be issues related to prescribing efficacy, and this warrants further consideration. This is particularly relevant when examining the prominence of levodopa as a cornerstone of therapeutic regimens for this disorder (see above).

That said, we wish to emphasise that our rationale for investigating prescribing practices related to Parkinson's disease was not driven by any perceived inefficiencies in clinical decision-making. Instead, our goal was to establish a clearly defined cohort of prescribers distributed across diverse geographical regions. This approach allows us to analyse prescribing patterns comprehensively and ensure that our findings are robust and reflective of varied practice settings. Ultimately, our study seeks to contribute to a better understanding of how treatments are prescribed and potentially pave the way for more informed discussions about optimising care for individuals living with neurodegenerative diseases.

To ensure that the readers are aware of this, we have revised the later discussion section of the report.

Likewise, there was no case made for the systematising or identification of key factors that should be considered when prescribing for PD other than it is not well understood.
Should be linked back to patient outcomes.

This point has now been addressed in our response above.

The TDF part of the work was: (a) not well explained, (b) not given a clear rationale for inclusion and (c) had underwhelming results.

Thank you for raising this point – as you can see from the revised paper, the inclusion of the TDF now sits within a much clearer narrative space and as such should be readily accessible for the readers of the journal (Table 1).

In the Methods, different sets of concepts were described which I found confusing at times – critical realism describing “mechanisms and contexts”, and theoretical domains describing behaviours and “behavioural determinants”.

Apologies for this. We have now ensured that the methodology section is much clearer to read by providing specific examples for each case.

I was very confused from this how the TDF was being used and on what data. How did you get from a list of factors to barriers to behaviour? What behaviour?

In the supplemental codebook, we provided examples of the key thematic data and their links to the Theoretical Domains Framework (TDF) model. This detailed information is intended to equip readers with sufficient context to assess the reliability and validity of our findings. To ensure readers are familiar with our specific application of the TDF, we followed the same methodological approach outlined in prior studies published in BMJ Open, which employed the TDF to identify behaviours that could facilitate key actions (e.g. Cahill *et al*. 2021). To enhance clarity, we have revised the manuscript to include additional narrative sections that explicitly guide readers to these previously published works. These references offer further insights into how the TDF has been used effectively in similar contexts, thereby strengthening the rationale for our approach.

By incorporating this expanded discussion, the revised manuscript makes the connection between the thematic data and the TDF framework more explicit. This addition ensures that readers can more easily understand how the TDF was applied to identify behaviours and mechanisms relevant to our study. Overall, these revisions aim to enhance the transparency and comprehensibility of our methodological choices and their alignment with established research practices (see ‘Data analysis’).

In the Table you have comments on the factors – are they what the participants said or what you interpreted from their comments or a mix of both? Is this reliable enough data?

As described earlier and further detailed in the methodology section of the report, the outcome of the NGT group is refined through a process of iterative discussion and consensus among the participants. During this process, specific statements made by individual group members are carefully examined and, where relevant, incorporated into broader thematic structures. These thematic structures, as represented in the tables, serve as a framework for organising and understanding the data. Subsequently, the group collectively ranks the relative importance of these behaviours, ensuring that the most significant aspects are prioritised.

To maintain the integrity and quality of the emergent thematic structure, every stage of this reduction process is conducted with the explicit agreement of the participants. At no point during this process does the facilitator impose a predefined analytic structure or direct the proceedings. Instead, the facilitator’s role is limited to guiding the discussion in a neutral manner, allowing the group to arrive at a consensus organically.

The outcome of this collaborative process is a set of factors that the group deems both relevant and critical to addressing the primary question posed by the key nominal prompt. To ensure transparency and to provide readers of the journal with a clear understanding of the internal validity and practical utility of this method, we have directed readers to additional methodological reports (see ‘Data analysis’).

The TDF does not map “determinants” as such but is used to categorise barriers to performing the defined target behaviour. You do not describe how you define the target behaviours. Are you saying each priority factor is linked to a different target behaviour? If so, how did you identify those behaviours? And what evidence do you have that these behaviours are not being done by the HCPs (implied by talk of intervention development)?

The TDF was developed by behavioural scientists and implementation researchers as a tool to consolidate various theories related to behaviour change into distinct, actionable domains. According to Atkins *et al*. (2017) and Clay *et al*. (2012), some of the key reports on the framework, this structured, theory-based approach facilitates the identification of determinants influencing behaviour.

The current paper builds on this earlier work.

The decision to sample this population stems from the ability to clearly define and access a discrete group of healthcare professionals (HCPs) working across Europe. This allowed us to design and test an approach that applies behavioural science principles to drive behaviour change effectively within this context. HCPs in the Parkinson’s disease space were identified as the ideal group for this effort, given the complexity of care required and the opportunity to address variability in prescribing practices.

This study represents an essential step within a broader framework, which is designed to be flexible and applicable across various disease states. The expanded narrative included in our work emphasises the universality of this approach, demonstrating its potential to understand and influence the target behaviour which, in this case, is prescribing in any clinical context.

Results of the TDF portion were underwhelming and contributed little to the field. Consider rewriting the paper to emphasize the ranked factors part of the work and 2consider submitting as a short report. I appreciate how much work went into the TDF section but it doesn't work here with your current data.

Thank you for raising this point, which we carefully considered during the revision process. Ultimately, we felt that the readership of the journal would gain more value from having access to the complete narrative of the study. By presenting the outcomes of the NGT discussions in conjunction with the broader TDF, we believe that the true impact and significance of the study can be fully understood and appreciated (see Figure 1, which makes the linkages between the NGT focus groups and the TDF model explicit).

To ensure that readers can effortlessly grasp the connection between the NGT results and the TDF framework, we have undertaken a comprehensive revision of the paper. Every section has been rewritten to enhance clarity, improve flow, and make the links between these critical components explicit. This approach not only strengthens the overall narrative but also ensures that the insights derived from the study are accessible and meaningful to the journal's audience.

References

Atkins L, Francis J, Islam R, *et al*. A guide to using the Theoretical Domains Framework of behaviour change to investigate implementation problems. *Implement Sci* 2017;12(1):1–18

Cahill LS, Carey LM, Mak-Yuen Y, *et al*. Factors influencing allied health professionals' implementation of upper limb sensory rehabilitation for stroke survivors: a qualitative study to inform knowledge translation. *BMJ Open* 2021;11(2):e042879

Cane J, O'Connor D, Michie S. Validation of the theoretical domains framework for use in behaviour change and implementation research. *Implement Sci* 2012;7:37

VERSION 2 - REVIEW

Reviewer 2

Name Long, Janet

Affiliation Australian Institute of Health Innovation, Australian Institute of Health Innovation

Date 17-Dec-2024

COI

Thanks for engaging with my comments and rewriting sections to improve the clarity. The links are clearer now. I have to admit I have only ever used TDF as a diagnostic tool - why aren't HCPs doing this recommended practice? How can we address the barriers? Therefore, I had expected the paper to be more in the form of here is a problem and here, after analysis are the interventions or strategies that can address the problem. Instead, the paper is saying, there is no problem here to fix as such, we are analyzing consensus practice to understand it better. The objectives do state this, so that is fair. However, if as in other papers the purpose is to offer prescribing support or interventions, then the discussion does end awkwardly with no suggestions offered and no description of the future steps of how to use this data. I suggest this would be a useful few sentences to add.

Figure 1 is still missing. Looks like it was not uploaded.

All the best for your future research.

VERSION 2 - AUTHOR RESPONSE

Response to Reviewers

R2 Thanks for engaging with my comments and rewriting sections to improve the clarity. The links are clearer now. I have to admit I have only ever used TDF as a diagnostic tool - why aren't HCPs doing this recommended practice? How can we address the barriers?

Therefore, I had expected the paper to be more in the form of here is a problem and here, after analysis are the interventions or strategies that can address the problem. Instead, the paper is saying, there is no problem here to fix as such, we are analyzing consensus practice to understand it better. The objectives do state this, so that is fair. However, if as in other papers the purpose is to offer prescribing support or interventions, then the discussion does end awkwardly with no suggestions offered and no description of the future steps of how to use this data. I suggest this would be a useful few sentences to add.

Thank you once again for your consideration of our work which again has allowed us an opportunity to improve our report. As you correctly highlighted our report was a description of research that we have carried out to understand the decision making that takes place during prescribing. It is important to further clarify that the work reported here is part of an on-going programme of research that serves to bring behavioural science strategies into the forefront of study of the prescription behaviour and triangulates three approaches to converge on a better understanding of decision making in this context. Please see our responses to your previous comments where such description is contained. As you indicated in the current comments, we also stated this in the objectives of the paper.

To clarify our report is not intended to 'to offer prescribing support or interventions' as you commented. Further to your suggestion we have now included a 'few sentences' towards the conclusion of the report to further clarify our aims (see final paragraph).

Figure 1 is still missing. Looks like it was not uploaded.

Thank you also for highlighting the omission of figure 1 – we can confirm that this has been submitted with the final revision.

All the best for your future research.

Thank you again for your ongoing support of our work.

VERSION 3 - REVIEW

**Reviewer**	**2**
**Name**	**Long, Janet**
**Affiliation**	**Australian Institute of Health Innovation, Australian Institute of Health Innovation**

Date **21-Jan-2025**

COI

Thanks for providing Figure 1. I suspect the journal will require a better image quality as it was hard to read but it's a useful addition to the paper.
